# Supplementary material for: The Phenotypic and Genetic Spectrum of Glycogen Storage Disease Type VI
Source: Genes (Basel). 2021 Aug 3;12(8):1205. doi: 10.3390/genes12081205 (PMC8391619; doi:10.3390/genes12081205)
Supplement: Supplementary file 1 [file genes-12-01205-s001.zip › Supplementary-latest version.pdf]

Supplemental Table S1: Publications included in the analysis.

1. Aeppli TR, Rymen D, Allegri G, Bode PK, Häberle J. Glycogen storage disease type VI: clinical course and molecular background. Eur J Pediatr. 2020;179:405–13.
2. Beauchamp NJ, Taybert J, Champion MP, Layet V, Heinz-Erian P, Dalton A, et al. High frequency of missense mutations in glycogen storage disease type VI. J Inherit Metab Dis. 2007;30:722–34.
3. Beyzaei Z, Ezgu F, Geramizadeh B, Imanieh MH, Haghighat M, Dehghani SM, et al. Clinical and genetic spectrum of glycogen storage disease in Iranian population using targeted gene sequencing. Sci Rep. 2021;11:7040.
4. Burwinkel B, Bakker HD, Herschkovitz E, Moses SW, Shin YS, Kilimann MW. Mutations in the liver glycogen phosphorylase gene (PYGL) underlying glycogenosis type VI. Am J Hum Genet. 1998;62:785–91.
5. Çakar NE, Gezdirici A, Topuz HŞ, Önal H. Novel variants in Turkish patients with glycogen storage disease. Pediatr Int. 2020;62:1145–50.
6. Chang S, Rosenberg MJ, Morton H, Francomano CA, Biesecker LG. Identification of a mutation in liver glycogen phosphorylase in glycogen storage disease type VI. Hum Mol Genet. 1998;7:865–70.
7. Davit-Spraul A, Piraud M, Dobbelaere D, Valayannopoulos V, Labrune P, Habes D, et al. Liver glycogen storage diseases due to phosphorylase system deficiencies: diagnosis thanks to non invasive blood enzymatic and molecular studies. Mol Genet Metab. 2011;104:137–43.
8. Eghbali M, Fatemi KS, Salehpour S, Abiri M, Saei H, Talebi S, et al. Whole-Exome Sequencing Uncovers Novel Causative Variants and Additional Findings in Three Patients Affected by Glycogen Storage Disease Type VI and Fanconi-Bickel Syndrome. Front Genet. 2020;11:601566.
9. Grünert SC, Rosenbaum-Fabian S, Hannibal L, Schumann A, Spiekerkoetter U. Successful pregnancy in a woman with glycogen storage disease type 6. Mol Genet Metab Rep. 2021;27:100770.
10. Grünert SC, Hannibal L, Schumann A, Rosenbaum-Fabian S, Beck-Wödl S, Haack TB, et al. Identification and Characterization of a Novel Splice Site Mutation Associated with Glycogen Storage Disease Type VI in Two Unrelated Turkish Families. Diagnostics (Basel). 2021;11.
11. Jagadisan B, Ranganath P. Glycogen Storage Disease Type VI With a Novel Mutation in PYGL Gene. Indian Pediatr. 2017;54:775–6.
12. Korula S, Danda S, Paul PG, Mathai S, Simon A. Hepatic Glycogenoses Among Children-Clinical and Biochemical Characterization: Single-Center Study. J Clin Exp Hepatol. 2020;10:222–7.
13. Liu B, Wu B, Lu Y, Zhang P, Xiao F, Li G, et al. A Novel, Recurrent, 3.6-kb Deletion in the PYGL Gene Contributes to Glycogen Storage Disease Type VI. J Mol Diagn. 2020;22:1373–82.
14. Lu S-Q, Feng J-Y, Liu J, Xie X-B, Lu Y, Abuduxikuer K. Glycogen storage disease type VI can progress to cirrhosis: ten Chinese patients with GSD VI and a literature review. J Pediatr Endocrinol Metab. 2020;33:1321–33.
15. Luo X, Hu J, Gao X, Fan Y, Sun Y, Gu X, et al. Novel PYGL mutations in Chinese children leading to glycogen storage disease type VI: two case reports. BMC Med Genet. 2020;21:74.
16. Ogawa A, Ogawa E, Yamamoto S, Fukuda T, Sugie H, Kohno Y. Case of glycogen storage disease type VI (phosphorylase deficiency) complicated by focal nodular hyperplasia. Pediatr Int. 2010;52:e150-153.
17. Roscher A, Patel J, Hewson S, Nagy L, Feigenbaum A, Kronick J, et al. The natural history of glycogen storage disease types VI and IX: Long-term outcome from the largest metabolic center in Canada. Mol Genet Metab. 2014;113:171–6.
18. Tang NLS, Hui J, Young E, Worthington V, To K-F, Cheung K-L, et al. A novel mutation (G233D) in the glycogen phosphorylase gene in a patient with hepatic glycogen storage disease and residual enzyme activity. Mol Genet Metab. 2003;79:142–5.
19. Vega AI, Medrano C, Navarrete R, Desviat LR, Merinero B, Rodríguez-Pombo P, et al. Molecular diagnosis of glycogen storage disease and disorders with overlapping clinical symptoms by massive parallel sequencing. Genet Med. 2016;18:1037–43.
20. Zhan Q, Lv Z, Tang Q, Huang L, Chen X, Yang M, et al. Glycogen storage disease type VI with a novel PYGL mutation: Two case reports and literature review. Medicine (Baltimore). 2021;100:e25520.
